# Safety and long-term prognosis of simultaneous versus staged resection in synchronous colorectal cancer with liver metastasis: a systematic review and meta-analysis

**DOI:** 10.1186/s40001-022-00937-z

**Published:** 2022-12-19

**Authors:** Shi-hao Wang, Lei Song, Ji-yan Tang, Wei-peng Sun, Zhen Li

**Affiliations:** 1 Department of General Surgery, The First Affiliated Hospital of Zhengzhou University, Zhengzhou, China; 2 Academy of Medical Science, Zhengzhou University, Zhengzhou, China; 3 Department of Colorectal and Anal Surgery, The First Affiliated Hospital of Zhengzhou University, No. 1, Jianshe Road, Zhengzhou, 450052 China

**Keywords:** Synchronous colorectal liver metastases, Simultaneous resection, Staged resection, Colorectal cancer, Meta-analysis

## Abstract

**Purpose:**

The optimal time point for surgical resection of synchronous colorectal liver metastases (SCLMs) is still controversial. This meta-analysis evaluated the safety and long-term prognoses of simultaneous and staged resection of SCLM to provide a reference for clinical selection.

**Methods:**

A systematic literature search for studies published by October 2022 was performed using PubMed, Web of Science, Embase, Scopus and Cochrane Library. The evaluated outcome parameters were total, gastrointestinal and hepatic complications, as well as perioperative mortality, intraoperative blood loss, total hospital stay, 5-year disease-free survival (DFS) and 5-year overall survival (OS).

**Results:**

This meta-analysis included 22 nonrandomised and one randomised study comprising 4862 patients. The patients undergoing simultaneous resection of SCLM had similar total (OR = 0.88, 95% CI [0.66–1.19], *P* = 0.409), gastrointestinal (OR = 1.19, 95% CI [0.89–1.59], *P* = 0.241) and hepatic (OR = 1.04, 95% CI [0.83–1.31], *P* = 0.734) complications, as well as perioperative mortality (OR = 1.79, 95% CI [0.88–3.64], *P* = 0.108), 5-year DFS (HR = 1.26, 95% CI [0.96–1.66], *P* = 0.098) and 5-year OS (HR = 1.13, 95% CI [0.95–1.34], *P* = 0.164). Lower intraoperative blood loss (SMD = − 0.39, 95% CI [− 0.60 to − 0.18], *P* < 0.001) and shorter total hospital stay (WMD = − 5.43, 95% CI [− 7.29 to − 3.58], *P* < 0.001) were observed in the simultaneous-resection group versus the staged group.

**Conclusions:**

Simultaneous resection is safe and effective for SCLM patients. The long-term prognosis is equivalent to that of the traditional staged resection. Correct selection of resectable SCLM patients for the simultaneous resection of the primary tumour and liver metastases can be the first choice. Owing to the potential heterogeneity, more RCTs should be included to verify our conclusions.

**Supplementary Information:**

The online version contains supplementary material available at 10.1186/s40001-022-00937-z.

## Introduction

Colorectal cancer (CRC) is one of the most common malignant tumours worldwide [1]. According to the global cancer statistics in 2020 [2], the incidence and mortality of colorectal cancer ranked third and second, respectively. Because most mesenteric blood flows back to the portal vein, the liver is the most common metastatic site of colorectal cancer [3]. In fact, the liver is the only site with distant metastasis in approximately 20% of patients [4]. Approximately 15–25% of patients with CRC also present with synchronous liver metastases [5], a condition called synchronous colorectal liver metastases (SCLM). Patients with CRC who develop liver metastases are at a higher risk of death; the median overall survival (OS) of patients who cannot undergo surgery is 6–12 months [6]. Patients with SCLM can only be treated by resecting the metastases, representing the only hope for survival and cure [7, 8].

According to the time point of the surgery, resection of liver metastases can be classified into simultaneous resection (simultaneous resection of the primary tumour and liver metastases) and staged resection (resection of the primary tumour first, followed by resection of the metastases after 3–6 months) [9, 10]. Traditional views have advocated the staged strategy because previous findings have shown that simultaneous resection is highly traumatic and associated with higher perioperative complications and mortality rates than staged resection [11, 12]. The staged strategy also enables the identification of the small metastatic lesions in the liver or elsewhere that may be occult, in addition to the primary lesion. It is also possible to control the tumours via chemotherapy during the observation period after primary-tumour resection, which can provide a basis for selecting the subsequent chemotherapy regimen [13, 14]. A considerable number of studies have shown that in recent years, with the improvements in surgical operations and perioperative care, the mortality and perioperative complications after simultaneous resection have not been significantly different from those observed after staged resection [15, 16]. In addition, simultaneous resection prevents the loss of surgical opportunity that may result from tumour progression, as well as the physical and psychological trauma that a secondary surgery can cause to the patient [13, 17]. However, there is currently no consensus on the timing of the surgery for liver metastases derived from synchronous colorectal cancer. Therefore, this study compared the outcome parameters of the simultaneous surgery with those of the staged surgery, thereby providing a reference for the choice of the timing of surgery in SCLM.

## Methods

PRISMA 2020 guidelines and AMSTAR guidelines were followed in conducting this meta-analysis [18]. The protocol has been registered on PROSPERO (CRD42021282727).

### Search strategy

The databases of PubMed, Web of Science, Embase, Scopus and Cochrane Library were carefully screened for studies published by October 2022 by using the following keywords: “colorectal neoplasms,” “colorectal tumours,” “colorectal cancers,” “liver metastases,” “synchronous colorectal liver metastases,” “simultaneous resection,” “synchronous resection,” “staged resection,” and “delayed resection.” This search strategy was slightly adjusted to comply with the different database requirements and the references of the relevant articles were screened for additional relevant studies.

### Inclusion and exclusion criteria

The inclusion criteria were defined based on the Population, Intervention, Comparison, Outcome and Study (PICOS) design principle as follows: (1) patients with SCLM (preoperatively or intraoperatively diagnosed with liver metastases; (2) both the primary colorectal tumour and liver metastases were resectable at the time of diagnosis; (3) the selected studies reported at least one outcome of interest; (5) when more than one report per study was available, the one with the best quality or published most recently was included in this analysis; (6) only randomised controlled trials (RCTs) and non-RCTs (nRCTs) published in English were included. Following were the exclusion criteria: (1) patients with extrahepatic metastases; (2) " liver first " resection (liver resection first, followed by primary-tumour resection); (3) studies lacking a control group or in which the control group was unreasonable; (4) a publication type which is not suitable (case reports, conference abstracts, meta-analyses, reviews and animals experiments); (5) reports not written in English; (6) low-quality studies; and (7) no original data could be obtained from the corresponding author.

### Data extraction

Literature review, data extraction and quality assessment were independently performed by two reviewers (Shi-hao Wang and Lei Song). A full reading of the text resolved all inconsistencies between the two reviewers. The outcomes we focussed on were complications (including total as well as gastrointestinal and hepatic complications), perioperative characteristics (including perioperative mortality, intraoperative blood loss and total hospital stay) and long-term outcomes (including 5-year disease-free survival [DFS] and 5-year overall survival [OS]). The DFS and OS were calculated since the hepatectomy. Some outcome measures could not be obtained directly from the text and thus the following definitions were made: (1) gastrointestinal complications comprised anastomotic leakage, bleeding, ileus, colitis and abdominal and pelvic abscesses; (2) hepatic complications comprised perihepatic or subdiaphragmatic abscess, right-sided pleural effusion, bile leak and/or biloma and hepatic insufficiency or failure.

### Quality evaluation

The Cochrane Risk of Bias Tool for Randomised Controlled Trials was used to evaluate the quality of the included RCT. nRCTs were evaluated using the Newcastle–Ottawa Quality Assessment Scale (NOS).

### Statistical methods

The statistical analyses were carried out using the STATA software, version 14.0. Continuous variables were analysed using weighted/standardised mean differences (WMD/SMD) and 95% confidence intervals (CIs). Dichotomous variables were analysed using odds ratios (ORs) and 95% CIs. If mean values or standard deviations (SDs) were not provided in a report, we calculated the SDs from the median values and ranges using Wan et al.’s method [19]. If hazard ratios (HRs) and 95% CIs were not reported, the method published by Tierney et al. [20] was used to estimate the HR as the effect indicator to pool the survival statistics. Heterogeneity was measured using Cochran's *Q* test and the Chi-square test. The results were presented with the corresponding 95% CIs and statistical significance was set at *P* < 0.05. The random-effects model should be used as a default due to clinical heterogeneity among surgical trials [21]. If the studies were significantly heterogeneous (*I*^2^ > 50%), a sensitivity analysis was conducted using the leave-one-out approach to check the stability of the meta-analysis. In addition, subgroup analyses were performed on studies with NOS scores ≥ 7 and studies with more than 50 patients in the simultaneous group to assess consistency in data reporting. Finally, the studies were assessed for any publication bias using funnel plots and Begg's test.

## Results

### The basic characteristics about the included studies

**Fig. 1:**
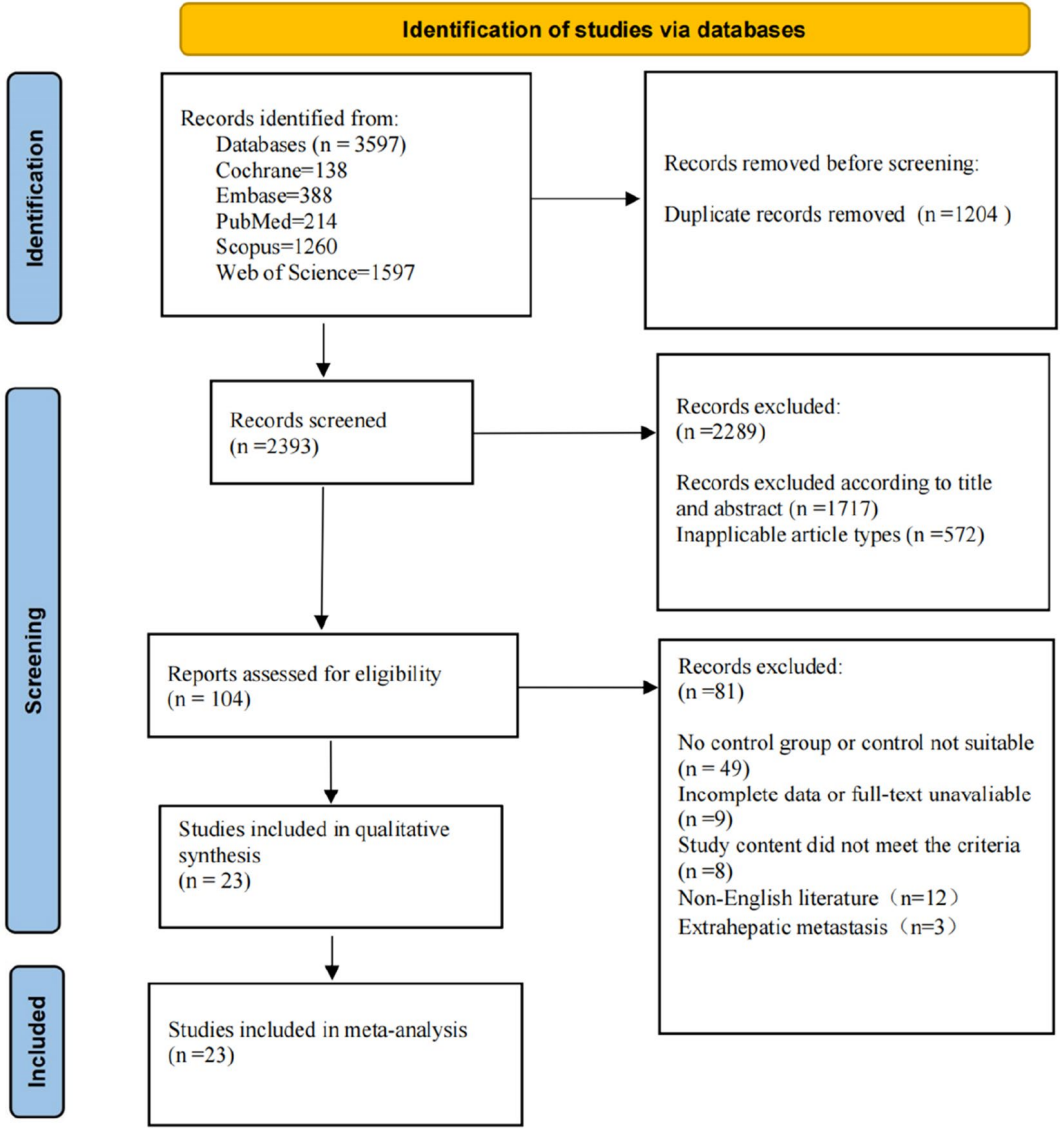
Process of study selection

**Fig. 2:**
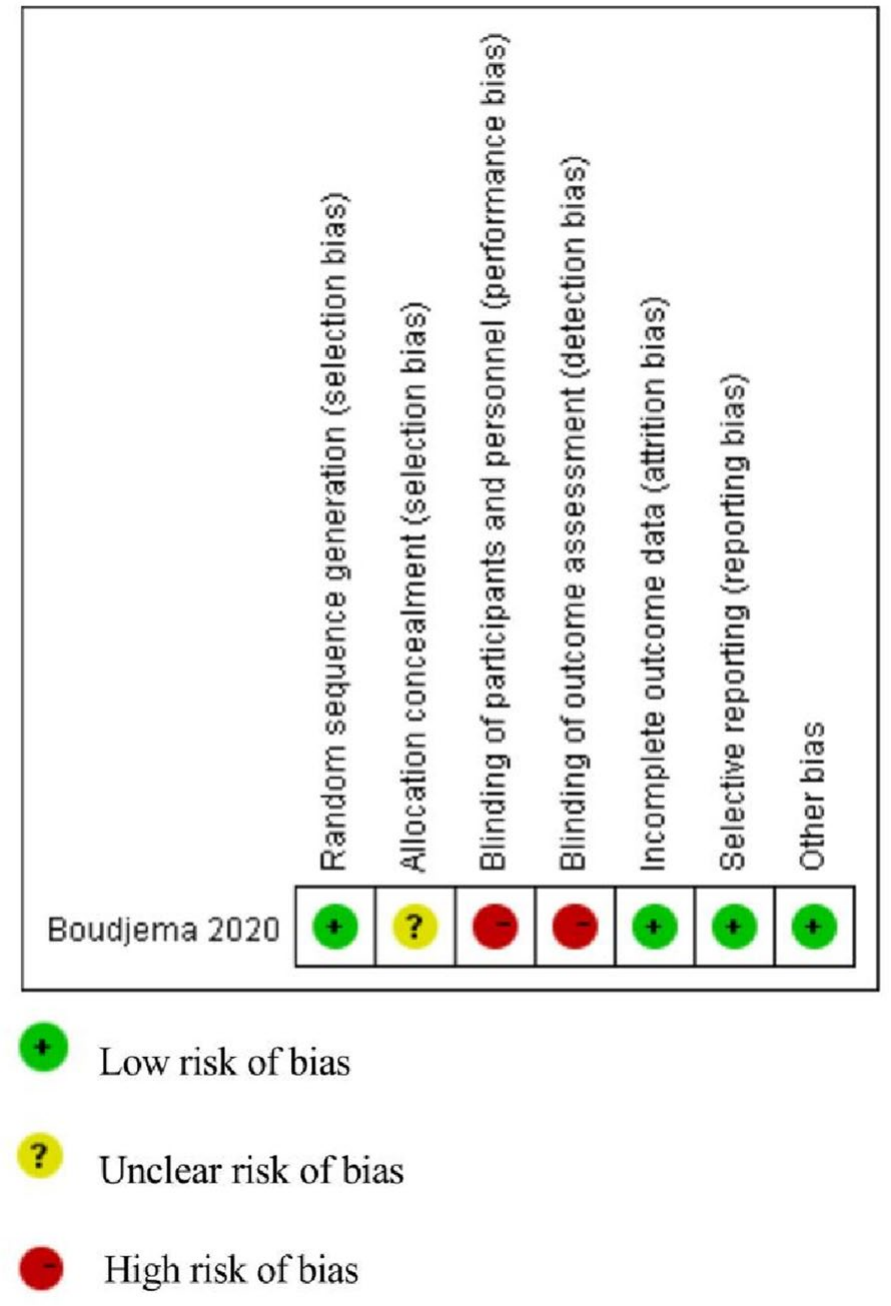
Methodological quality of the randomised controlled trial

A total of 3597 publications were retrieved from the 5 databases and 23 of them were found eligible according to the specified inclusion and exclusion criteria (Fig. 1). In total, only 1 RCT [22] and 22 nRCTs [13, 14, 17, 23–41], comprising a total of 4862 patients (2056 and 2806 cases of simultaneous resection and staged resection, respectively) were included. All the nRCTs had a NOS score ≥ 6 and the RCT had a low bias risk, assessed using RevMan 5.3 (Fig. 2). The basic characteristics of the patients in the included studies are shown in Table 1.

**Table 1:** The basic characteristics of the included studies

First author	Year	Region	Study design	Number of patients		Age^a^		Gender (M:F)		NOS
				Simultaneous resection	Staged resection	Simultaneous resection	Staged resection	Simultaneous resection	Staged resection	
Valdimarsson	2020	Sweden	nRCT	160	377	65 (58–72)	66 (58–73)	(90:70)	(234:143)	6
Kaibori	2010	Japan	nRCT	32	42	62.3 ± 9.3	65.0 ± 9.9	(17:15)	(27:15)	7
Le Souder	2018	Canada	nRCT	26	27	54.7 ± 12.9	58.7 ± 9.2	(14:12)	(12:15)	7
Luo	2010	China	nRCT	129	276	58 (42–69)	60 (43–70)	(76:53)	(156:120)	6
Martin	2009	USA	nRCT	70	160	58	61	(54:46)	(57:43)	6
Martin	2003	USA	nRCT	134	106	64 (27–85)	61 (23–82)	(69:65)	(61:45)	7
Moug	2010	United Kingdom	nRCT	32	32	69 (53–79)	67 (37–82)	(18:14)	(21:11)	6
She	2014	China	nRCT	28	88	65.5 (29–75)	59 (24–81)	(22:6)	(59:29)	8
Thelen	2007	Germany	nRCT	40	179	60.5 ± 13.4	59.7 ± 10.7	(24:16)	(96:84)	8
Tsilimigras	2021	USA, Italy, Romania	nRCT	314	379	61 (51–69)	59 (51–66)	(189:125)	(224:152)	6
Turrini	2007	France	nRCT	57	62	60	59	(48:9)	(51:11)	7
Weber	2003	France	nRCT	35	62	58 ± 12	60 ± 9	(18:17)	(31:31)	7
Yan	2007	Australia	nRCT	73	30	60 ± 10	59 ± 13	(33:40)	(15:15)	7
Vassiliou	2007	Greece	nRCT	25	78	63 ± 12	61 ± 14	(15:10)	(47:31)	6
de Haas	2010	France	nRCT	26	26	60 ± 8	60 ± 8	(17:9)	(17:9)	7
Boudjema	2020	France	RCT	39	46	68 ± 9	66 ± 11	(25:14)	(25:21)	/
Kye	2019	Korea	nRCT	143	65	≤ 65(93:51)	> 65(50:14)	(102:41)	(43:22)	7
Abbott	2012	USA	nRCT	60	84	57.5 (45.5–63.9)	53.3 (45.5–60.8)	(40:20)	(49:35)	8
Capussotti	2006	Italy	nRCT	31	48	62.4 (49–78)	59.3 (31–77)	(18:13)	(27:21)	7
Chua	2004	USA	nRCT	64	32	63 ± 11	61 ± 12	(39:25)	(18:14)	6
Wu	2022	China	nRCT	495	495	57.2 ± 11.4	57.3 ± 11.2	(296:199)	(293:202)	6
Thongkan	2020	Thailand	nRCT	21	90	56.4 ± 12.6	61.3 ± 11.5	(13:8)	(47:43)	7
Karam	2022	France	nRCT	22	22	65 (60–77)	59 (52–65)	(20:2)	(14:8)	7

*nRCT* non-randomised controlled trial, *RCT* randomised controlled trial, *M* male, *F* female

^a^Mean ± SD, median (range), or parameter (participants of simultaneous resection: participants of staged resection)

### The tumour characteristics in the included studies

We analysed the tumour characteristics, including whether neoadjuvant chemotherapy was administered, the sizes and number of metastases, unilobar or bilobar distribution and the number of metastases that required major resection (≥ 3 segments) or minor resection (< 3 segments), in the included studies. The rate of neoadjuvant chemotherapy was higher in the staged-resection group than in the simultaneous-resection group and the patients in the staged-resection group were more likely to undergo major resection of liver metastases than those in the simultaneous-resection group. These differences were all statistically significant. The remaining indicators, such as the sizes and number of metastases with unilobar or bilobar distribution, were not significantly different between the two groups. Table 2 shows the tumour characteristics in the included studies.

**Table 2:** The tumour characteristics in the included studies

First author	Year	Neoadjuvant chemotherapy		The size of the metastases^a^		The number of the metastases^a^		
		Simultaneous resection	Staged resection	Simultaneous resection	Staged resection	Simultaneous resection	Staged resection	
Valdimarsson	2020	101 (63.13%)	274 (72.68%)	20 (12–30) mm	20 (14–35) mm	2 (1–4)	2 (1–4)	
Kaibori	2010	/	17 (40.48%)	/	/	1: 2: 3 (4: 20: 8)	1: 2: 3 (9: 11: 22)	
Le Souder	2018	/	/	2 (1–3) cm	3 (2–6) cm	4 (2–8)	3 (1–4)	
Luo	2010	51 (39.53%)	169 (61.23%)	< 5 cm: ≥ 5 cm (76: 53)	< 5 cm: ≥ 5 cm (153: 123)	1: > 1 (81: 48)	1: > 1 (97: 179)	
Martin	2009	22 (31.43%)	130 (81.25%)	3.7 (0.3–8.8) cm	4 (0.8–13.0) cm	3 (1–16)	3 (1–8)	
Martin	2003	/	/	≤ 5 cm: > 5 cm (103: 31)	≤ 5 cm: > 5 cm (65: 41)	1: > 1 (81: 53)	1: > 1 (42: 64)	
Moug	2010	/	/	/	/	/	/	
She	2014	4 (14.29%)	28 (31.82%)	3.25 (1–20) cm	3.25 (0.8–21) cm	2 (1–multiple)	2 (1–multiple)	
Thelen	2007	/	/	≤ 50 mm: > 50 mm (24: 16)	≤ 50 mm: > 50 mm (101: 78)	1–3: ≥ 4 (34: 6)	1–3: ≥ 4 (122: 57)	
Tsilimigras	2021	80 (25.48%)	130 (34.30%)	2.5 (1.5–4.1) cm	3.0 (1.9–4.8) cm	2 (1–3)	2 (1–4)	
Turrini	2007	/	/	/	/	< 4: > 4 (42: 15)	< 4: > 4 (27: 35)	
Weber	2003	/	/	≤ 5 cm: > 5 cm (25: 10)	≤ 5 cm: > 5 cm (38: 24)	1–3: ≥ 4 (29: 6)	1–3: ≥ 4 (39: 23)	
Yan	2007	/	/	3.8 ± 3.4 cm	5.9 ± 3.8 cm	4 ± 2	3 ± 2	
Vassiliou	2007	/	/	/	/	/	/	
de Haas	2010	8(30.77%)	24(92.31%)	38 ± 33 mm	41 ± 21 mm	1: 2–3: > 3 (15: 7: 4)	1: 2–3: > 3 (15: 7: 4)	
Boudjema	2020	24 (61.54%)	28 (60.87%)	40.8 ± 28.8 mm	37.2 ± 33.5 mm	2 (1–13)	2 (1–9)	
Kye	2019	26 (18.18%)	35 (53.85%)	2.8 ± 1.9 cm	2.7 ± 2.1 cm	1.6 ± 1.1	2.4 ± 2.0	
Abbott	2012	46 (76.67%)	52 (61.90%)	/	/	≤ 5: > 5 (55: 5)	≤ 5: > 5 (57: 27)	
Capussotti	2006	3 (9.68%)	38 (79.17%)	5.5 (2.5–20) cm	5 (0.6–14) cm	1: 2: 3: 4: ≥ 5 (15: 5: 3: 4: 4)	1: 2: 3: 4: ≥ 5 (13: 12: 7: 6: 10)	
Chua	2004	/	/	3.7 ± 3.4 cm	3.9 ± 2.8 cm	2.5 ± 2.6	3 ± 2.2	
Wu	2022	213(43.03%)	202(40.81%)	/	/	/	/	
Thongkan	2020	8(38.10%)	84(93.33%)	40.1 (7.0–160.0) mm	41.5 (8.0–165.0) mm	2.4 (1.0–11.0)	2.4 (1.0–10.0)	
Karam	2022	19(86.36%)	17(77.27%)	24 (16–41) mm	20 (10–39) mm	5 (3–6)	3 (2–6)	
Pooled differences (95% CI)		0.33(0.19–0.56) *P* < 0.001		–0.10 (− 0.23 to 0.03) *P* = 0.123		0.17 (− 0.25 to 0.58) *P* = 0.437		

First author	Unilobar distribution		Bilobar distribution		Major resection		Minor resection	
	Simultaneous resection	Staged resection	Simultaneous resection	Staged resection	Simultaneous resection	Staged resection	Simultaneous resection	Staged resection
Valdimarsson	/	/	/	/	25 (15.63%)	152 (40.32%)	135 (84.37%)	222 (59.68%)
Kaibori	25 (78.13%)	22 (52.38%)	7 (21.88%)	20 (47.62%)	/	/	/	/
Le Souder	11 (42.31%)	13 (48.15%)	15 (57.69%)	14 (51.85%)	12 (46.15%)	12 (44.44%)	14 (53.85%)	15 (55.56%)
Luo	/	/	/	/	44 (34.11%)	133(48.19%)	85(65.89%)	143(51.81%)
Martin	/	/	/	/	/	/	/	/
Martin	/	/	/	/	/	/	/	/
Moug	/	/	/	/	/	/	/	/
She	/	/	8 (28.57%)	24 (27.27%)	12(42.86%)	54(61.36%)	16(57.14%)	34(38.64%)
Thelen	25 (62.50%)	91 (50.84%)	15 (37.50%)	88 (49.16%)	15 (37.50%)	142 (79.33%)	25 (62.50%)	37 (20.67%)
Tsilimigras	/	/	/	/	61(19.43%)	164(43.27%)	253(80.57%)	212(55.94%)
Turrini	43 (75.44%)	25 (40.32%)	14 (24.56%)	37 (59.68%)	/	/	/	/
Weber	27 (77.14%)	20 (32.26%)	8 (22.86%)	42 (67.74%)	11 (31.43%)	35 (56.45%)	24 (68.57%)	27 (43.55%)
Yan	23 (31.51%)	20 (66.67%)	50 (68.49%)	10 (33.33%)	/	/	/	/
Vassiliou	/	/	5 (20.00%)	17 (21.79%)	7 (28.00%)	23 (29.49%)	18 (72.00%)	55 (70.51%)
de Haas	19 (73.08%)	19 (73.08%)	7 (26.92%)	7 (26.92%)	/	/	/	/
Boudjema	23 (58.97%)	27 (58.70%)	16 (41.03%)	19 (41.30%)	/	/	/	/
Kye	/	/	/	/	/	/	/	/
Abbott	/	/	/	/	20 (33.33%)	63 (75.00%)	40 (66.67%)	21 (25.00%)
Capussotti	/	/	10 (32.26%)	18 (37.50%)	/	/	/	/
Chua	32 (50.00%)	11 (34.38%)	32 (50.00%)	21 (65.63%)	/	/	/	/
Wu	307 (62.02%)	294 (59.39%)	188 (37.98%)	201 (40.61%)	99 (20.00%)	106 (21.41%)	396 (80.00%)	389 (78.59%)
Thongkan	/	/			2 (9.52%)	19 (21.11%)		
Karam	/	/	9(40.91%)	13(59.09%)	2(9.09%)	1(4.55%)	20 (90.91%)	21 (95.45%)
	1.52 (0.90–2.58) *P* = 0.117		0.69 (0.46–1.03) *P* = 0.070		0.43 (0.29–0.64) *P* < 0.001		2.34 (1.53–3.58) *P* < 0.001	

### Complications

#### Total complications

**Fig. 3:**
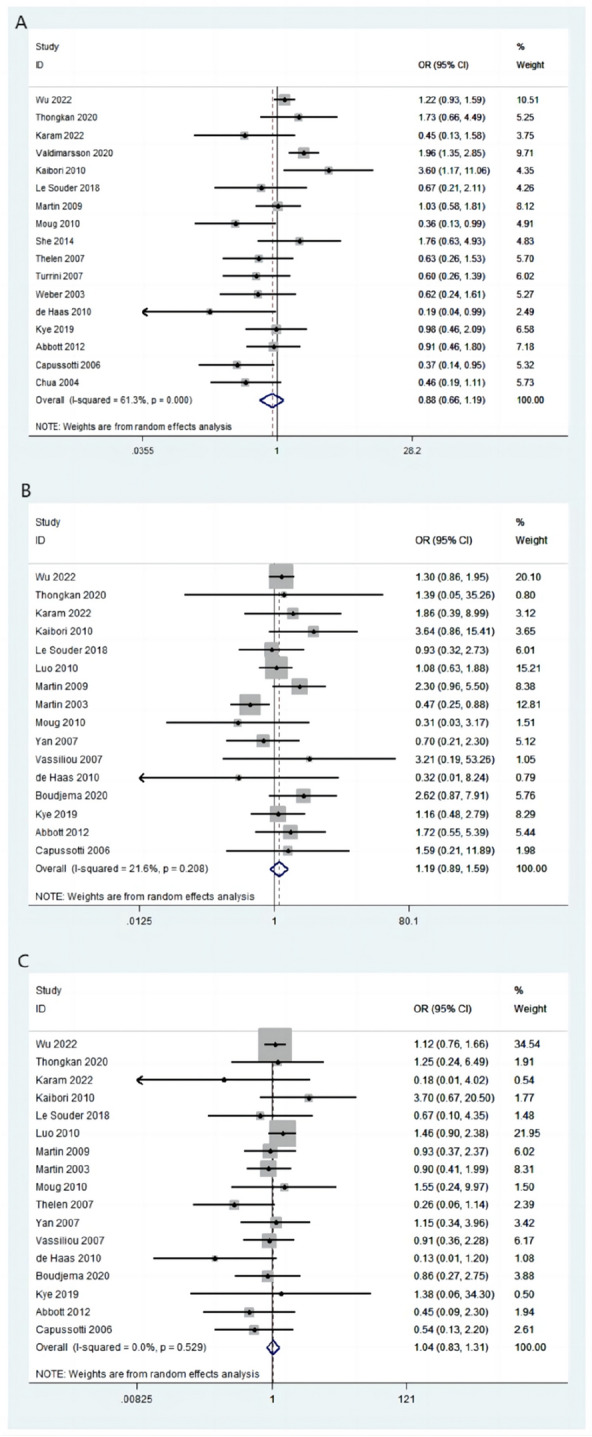
Complications between two groups shown as a forest plot. **A** Total complications. **B** Gastrointestinal complications. **C** Hepatic complications

A total of 17 articles described total complications; 479/1342 (35.69%) patients in the simultaneous-resection group and 667/1891 (35.27%) patients in the staged-resection group had total complications. Total complication rates between the two groups were not significantly different (OR = 0.88, 95% CI [0.66–1.19], *P* = 0.409; *I*^2^ = 61.3%; Fig. 3A).

#### Gastrointestinal complications

Gastrointestinal complications were reported in 16 articles. In the simultaneous- and staged-resection groups, gastrointestinal complications occurred in 188/1358 (13.84%) and 186/1627 (11.43%) patients, respectively. There were no statistically significant differences between the two groups in terms of gastrointestinal complications (OR = 1.19, 95% CI [0.89–1.59], *P* = 0.208; *I*^2^ = 21.6%; Fig. 3B).

#### Hepatic complications

There were 17 publications describing hepatic complications, which occurred in 166/1398 (11.87%) patients in the simultaneous-resection group and 252/1806 (13.95%) patients in the staged-resection group. There was no statistically significant difference in hepatic complication rate between the two groups (OR = 1.04, 95% CI [0.83–1.31], *P* = 0.734; *I*^2^ = 0.0%; Fig. 3C).

### Perioperative characteristics

#### Perioperative mortality

**Fig. 4:**
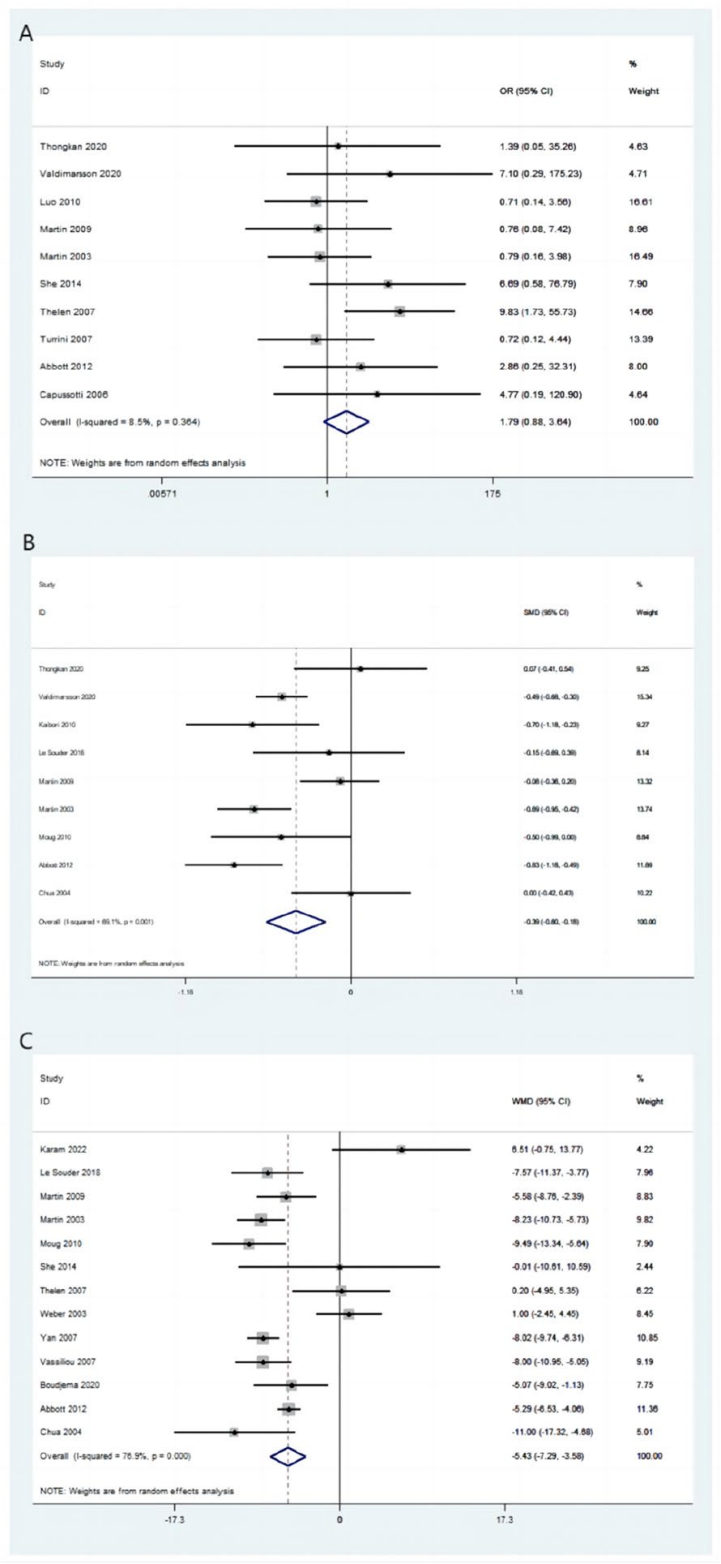
Forest plot of perioperative characteristics between two groups. **A** Perioperative mortality. **B** Intraoperative blood loss. **C** Total hospital stay

Perioperative mortality was reported in 10 publications. Based on the pooled outcome analysis, perioperative mortality was reported in 18/730 (2.47%) patients in the simultaneous-resection group and 20/1470 (1.36%) patients in the staged-resection group. Perioperative mortality did not significantly differ between the two groups (OR = 1.79, 95% CI [0.88–3.64], *P* = 0.108; *I*^2^ = 8.5%; Fig. 4A).

#### Intraoperative blood loss

A total of 10 publications reported intraoperative blood loss and the heterogeneity substantially decreased (from 91.4% to 69.1%) after excluding one publication [30] and thus 9 publications were included in the pooled analysis. The incidence of intraoperative blood loss in the simultaneous-resection group was significantly lower than that in the staged-resection group (SMD = –0.39, 95% CI [− 0.60 to − 0.18], *P* < 0.001; *I*^2^ = 69.1%; Fig. 4B).

#### Total hospital stay

Total hospital stay was reported in 14 articles and the heterogeneity substantially decreased (from 94.3% to 76.9%) after excluding one publication[40] and thus 13 publications were included in the pooled analysis. The average total hospital stay was five days shorter in the simultaneous resection than in the staged-resection group (WMD = –5.43, 95% CI [− 7.29 to − 3.58], *P* < 0.001; *I*^2^ = 76.9%; Fig. 4C).

### Long-term prognosis

#### The 5-year DFS

**Fig. 5:**
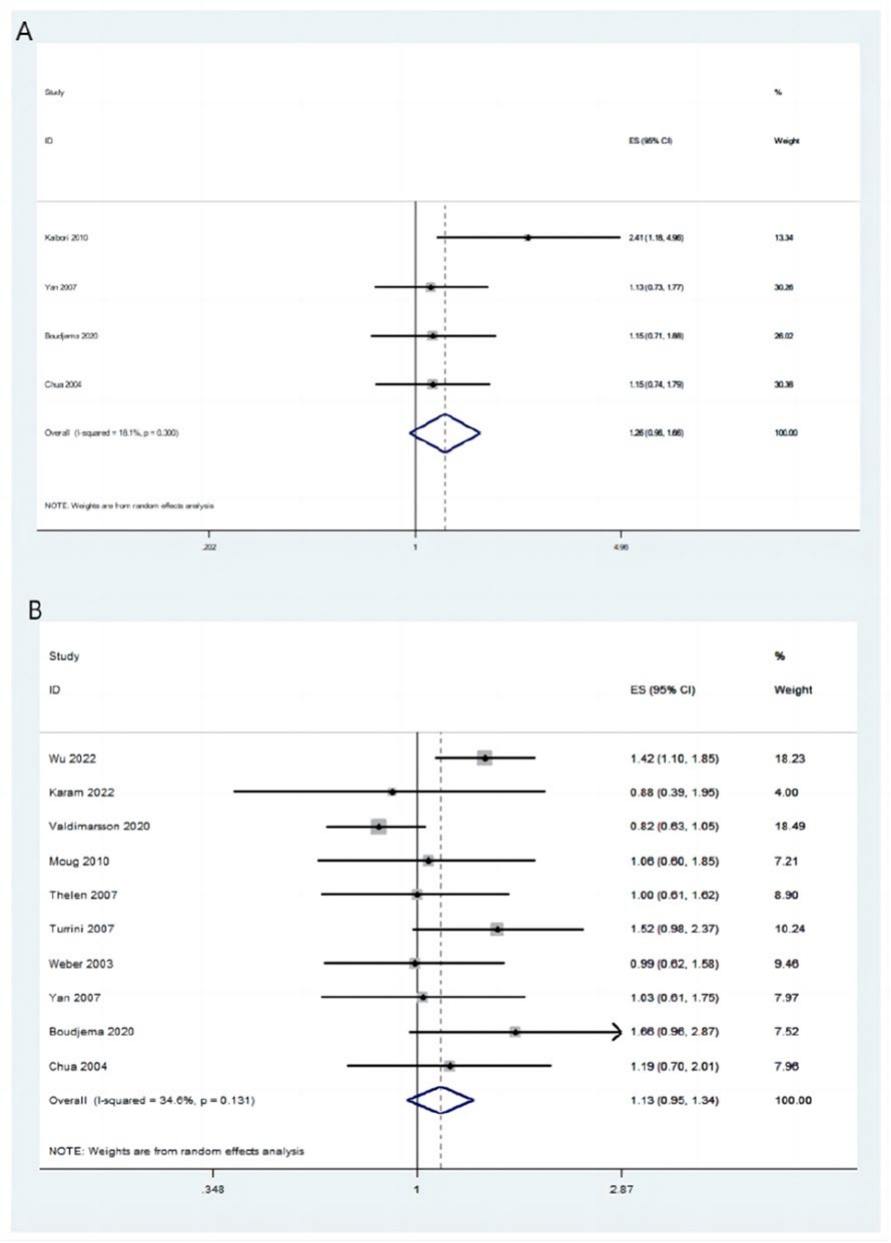
The forest plot compares long-term prognosis between two groups. **A** The 5-year DFS. **B** The 5-year OS

A total of 4 publications reported the 5-year DFS, which was not significantly different between the simultaneous- and staged-resection groups (HR = 1.26, 95% CI [0.96–1.66], *P* = 0.098; *I*^2^ = 18.1%; Fig. 5A).

#### The 5-year OS

A total of 10 publications reported the 5-year OS, which was not significantly different between the simultaneous- and staged-resection groups (HR = 1.13, 95% CI [0.95–1.34], *P* = 0.164; *I*^2^ = 34.6%; Fig. 5B).

Each outcome is listed in Table 3.

**Table 3:** Pooled results of the comparison of all outcomes between the two groups

Outcomes	Number of studies	Number of patients		WMD/SMD/OR/HR	95% CI	Heterogeneity	*P* value
		Simultaneous resection	Staged resection				
Complications							
Total complications	17	1342	1891	OR = 0.88	0.66–1.19	*I*^2^ = 61.3%, *P* < 0.001	*P* = 0.409
Gastrointestinal complications	16	1358	1627	OR = 1.19	0.89–1.59	*I*^2^ = 21.6%, *P* = 0.208	*P* = 0.241
Hepatic complications	17	1398	1806	OR = 1.04	0.83–1.31	*I*^2^ = 0.0%, *P* = 0.529	*P* = 0.734
Perioperative characteristics							
Perioperative mortality	10	730	1470	OR = 1.79	0.88–3.64	*I*^2^ = 8.5%, *P* = 0.364	*P* = 0.108
Intraoperative blood loss	9	599	950	SMD = –0.39	–0.60 to –0.18	*I*^2^ = 69.1%, *P* = 0.001	*P* < 0.001
Total hospital stay	13	648	946	WMD = –5.43	–7.29 to –3.58	*I*^2^ = 76.9%, *P* < 0.001	*P* < 0.001
Long-term prognosis							
5-year DFS	4	208	150	HR = 1.26	0.96–1.66	*I*^2^ = 18.1%, *P* = 0.300	*P* = 0.098
5-year OS	10	1017	1337	HR = 1.13	0.95–1.34	*I*^2^ = 34.6%, *P* = 0.131	*P* = 0.164

### Subgroup analysis

#### *NOS score* ≥ *7*

Subgroup analysis of studies with NOS score ≥ 7 found that the intraoperative blood loss (SMD = − 0.50, *P* = 0.002) and total hospital stay (WMD = − 4.87, *P* < 0.001) were reduced in the simultaneous group, but there were no differences in total complications (OR = 0.82, *P* = 0.307), gastrointestinal complications (OR = 0.99, *P* = 0.973), hepatic complications (OR = 0.74, *P* = 0.207), perioperative mortality (OR = 2.29, *P* = 0.067), 5-year DFS (HR = 1.56, *P* = 0.234) and 5-year OS (HR = 1.11, *P* = 0.366).

#### More than 50 patients in the simultaneous group

Subgroup analysis was performed on the studies with more than 50 simultaneous group participants. The summary results showed that the intraoperative blood loss (WMD = − 239.90, *P* < 0.001) and the total hospital stay (WMD = − 1.17, *P* < 0.001) were reduced in the simultaneous group, but there were no differences in the total complications (OR = 1.05, *P* = 0.769), gastrointestinal complications (OR = 1.09, *P* = 0.673), hepatic complications (OR = 1.14, *P* = 0.315), perioperative mortality (OR = 1.00, *P* = 0.990), 5-year DFS (HR = 1.14, *P* = 0.412) and 5-year OS (HR = 1.16, *P* = 0.302). The results of all subgroup analyses are summarised in Additional file 1: Table S1.

### Sensitivity analysis

**Fig. 6:**
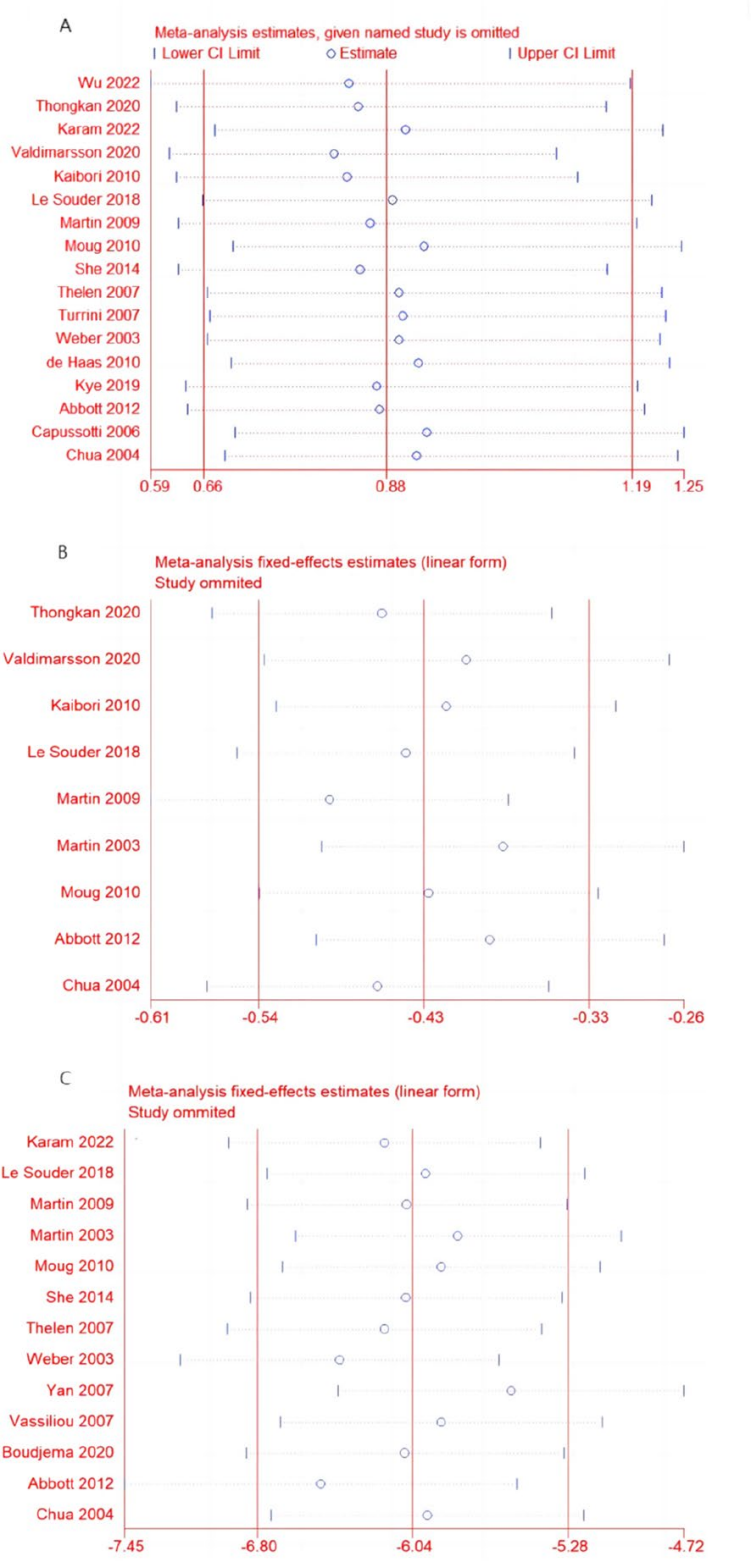
Sensitivity analysis. **A** Total complications. **B** Intraoperative blood loss. **C** Total hospital stay

Sensitivity analysis was carried out using the leave one-out approach. This analysis indicated that exclusion of any single study did not significantly affect the pooled results. The results of the meta-analysis were therefore concluded to be stable and reliable (Fig. 6).

### Publication bias

**Fig. 7:**
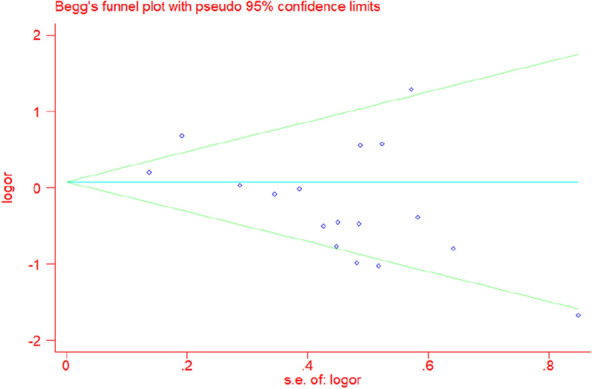
Funnel plot of the total postoperative complications (Begg's test, *P* = 0.149)

We assessed the funnel plot of the total complications for any publication bias. The funnel plot was found to be symmetrical, indicating a lack of publication bias (Fig. 7).

## Discussion

It is estimated that 50% of patients with CRC develop liver metastases [42]. The current treatments for SCLM include surgical resection, radiofrequency ablation, cryosurgery, hepatic arterial infusion and systemic chemotherapy [43]. Although the combination of chemotherapy, molecular targeted therapy and radiotherapy prolongs the median survival time to 24 months [30], the 5-year OS rate of nonsurgically treated patients remains low [44]. Resection of liver metastases caused by colorectal cancer has been shown to be effective [45, 46]. However, it is still controversial whether simultaneous or staged resection is indicated for SCLM and no consensus regarding the surgery indications or timing has been reached yet [30, 38].

Several authors have reported that simultaneous resection is associated with poorer short-term outcomes than staged resection [47, 48]. However, several studies have also recently shown that simultaneous colorectal and liver surgery is feasible and safe [31, 32]. Growing evidence indicates that there is no significant difference in postoperative complication rate or perioperative mortality between simultaneous resection and staged resection of SCLMs [29, 34, 38]. Haas et al. [26] have analysed 228 patients with SCLM and observed a decreasing trend in the incidence of total postoperative complications when the liver metastases were removed via simultaneous resection instead of staged resection and there was no statistically significant difference between the two procedures in terms of perioperative mortality. Chua et al. [25] have reported that there is no significant difference between simultaneous and staged hepatectomy in terms of intraoperative blood loss, incidence of total postoperative complications, operative mortality, OS and length of hospital stay. Multiple scholars believe that simultaneous resection can impair the gastrointestinal function because the cumulative trauma of the two major surgeries in the intestine and liver causes the intestinal anastomoses to heal poorly and may even lead to anastomotic leakage [33]. Our pooled results showed no statistical difference between simultaneous resection and staged resection in terms of total, gastrointestinal and hepatic complications. Moreover, our results showed that simultaneous resection does not increase the complication rate, especially the rate of gastrointestinal complications or perioperative mortality.

The studies included in previous meta-analyses [15, 49] were all nRCTs and it is reassuring that our meta-analysis included the first RCT [22] to date on the surgery timing for SCLMs, thus adding credence to our conclusions. In this study, we analysed 85 patients ([39 and 46] in the simultaneous- and staged-resection groups, respectively) and found that the rates of colonic, hepatic and general complications and perioperative mortality were [28.2% and 13.0%], [15.4% and 17.4%], [12.8% and 23.9%] and [7.4% and 3.2%], respectively. The above outcomes were not statistically different between the two groups. Importantly, we found that the simultaneous-resection group showed significantly shorter total hospital stay and lower incidence of intraoperative blood loss than the staged-resection group, consistent with the results of previously published meta-analyses. In addition, simultaneous resection prevents the opportunity of surgical treatment from being missed due to tumour progression, eliminates the pain associated with a second open surgery and allows early surgical adjuvant chemotherapy [17, 35].

We also compared the 5-year DFS and OS rates of the two procedures to evaluate the long-term prognoses and there was no statistically significant difference between the two groups. The result was satisfactory from a clinical standpoint. Although synchronous metastases have been shown to have a negative prognostic value [50], they are not a contraindication to hepatectomy if a resection that can cure the cancer is possible [51, 52]. Thus, simultaneous resection is safe and feasible for patients with resectable SCLM who can tolerate the surgery and have no extrahepatic metastasis [10].

The advancements in imaging technologies in recent years have enabled on-time detection of early, isolated or small liver metastases [53]. With the continuous improvements in surgical technologies, anaesthetic approaches, and perinatal treatments, the safety of simultaneous hepatectomy to treat SCLM has been dramatically improved, which has increasingly been recognised by scholars [54, 55]. A few authors still argue that a 2–6-month waiting period between the resection of the primary tumour and liver resection is necessary for the presentation of any subclinical metastasis, thereby enabling complete tumour clearance [56]. According to a cascade hypothesis, metastases develop in discrete steps, first in the liver and then in the lungs [57]. Thus, it seems too risky to wait for several months after the resection of the primary tumour. It is worth noting that after analysing the tumour characteristics of the two groups in our analysis, we found differences between the two groups in terms of the method used for metastasis removal. The staged-resection group had more cases of major resection of liver metastasis than the simultaneous-resection group. This observation indicates that patients with complex liver tumours require major resection and are more likely to undergo staged resection than simultaneous resection and also emphasises the importance of patient selection [17]. Although such selection bias may be reasonable, it may reduce the credibility of the evidence.

Our subgroup analyses showed consistency in the results, based on our results and previous studies, the optimum treatment for simultaneous liver metastasis should be based on the symptoms and general conditions of the patient, tumour location and degree, and whether there are other potential systemic diseases [29]. Staged hepatectomy can be considered for patients with advanced colorectal cancer who cannot tolerate surgery or have extrahepatic metastasis [28]. It is still noteworthy that minimally invasive surgery, represented by laparoscopic surgery, has been the main direction established in terms of surgical development in the future [58, 59]; the application of laparoscopic surgery for resection in CRLM has increased recently. Multiple retrospective studies [60–62] have shown a laparoscopic approach for CRLM to be safe, feasible and oncologically efficient when compared with traditional laparotomy. Although the laparoscopic resection for CRLM has made significant progress in the past 20 years, it is still an operation with high difficulty. In the process of laparoscopic resection for CRLM, the indications should be strictly followed and the laparotomy should be converted in time if necessary. Therefore, laparoscopic resection for CRLM is a safe and feasible option in qualified medical units. CRLM-related prognostic biomarkers have attracted increasing attention as a means of predicting prognosis. In some studies [63, 64], the preoperative lymphocyte-to-monocyte ratio (LMR) correlates accurately with clinical outcomes in patients with CRLM undergoing hepatic resection. However, the included studies need more data to facilitate such an analysis.

There are several limitations in the present study. First, the majority of the included studies were nRCTs and only one RCT was included, which might affect the quality of the data. Second, the tumour characteristics in each group were not exactly the same. Third, the lack of individual data from each study does not allow in-depth analyses. Although we used the random-effect model instead of the fixed-effect model, we still cannot exclude any possibility of bias. Nevertheless, only one RCT study is still meaningful and provides a valuable reference for our summary results.

## Conclusion

For the treatment of SCLM, simultaneous colectomy and hepatectomy are as safe as the staged approach. It does not increase the number of postoperative complications or perioperative mortality and can shorten the hospital stay and reduce the incidence of intraoperative bleeding. In terms of long-term prognosis, simultaneous resection is equivalent to staged resection. Therefore, simultaneous resection can be considered the first choice for the resection of the primary tumour and liver metastasis, provided that patients with resectable SCLM are carefully selected and the operation is performed by experienced staff.

## Supplementary Information

**Additional file 1: Table S1.** The results of all subgroup analyses.

## Data Availability

Data and materials will be available upon request.
