# Supplementary material for: Safety and long-term prognosis of simultaneous versus staged resection in synchronous colorectal cancer with liver metastasis: a systematic review and meta-analysis
Source: Eur J Med Res. 2022 Dec 19;27:297. doi: 10.1186/s40001-022-00937-z (PMC9762091; doi:10.1186/s40001-022-00937-z)
Supplement: Supplementary file 1 — Additional file 1: Table S1. The results of all subgroup analyses. [file 40001_2022_937_MOESM1_ESM.docx]

| Outcomes | Number of studies | WMD/SMD/OR/HR | 95% CI | Heterogeneity | *P*-value |
| --- | --- | --- | --- | --- | --- |
|  |  |  |  |  |  |
| **NOS score ≥7** |  |  |  |  |  |
| Total complications | 12 | OR = 0.82 | 0.57-1.19 | I^2^ = 43.1%, *P* =0.055 | *P* = 0.307 |
| Gastrointestinal complications | 10 | OR = 0.99 | 0.64-1.53 | I^2^ =18.0%, *P* = 0.278 | *P* = 0.973 |
| Hepatic complications | 11 | OR = 0.74 | 0.47-1.18 | I^2^ = 3.8%, *P* = 0.407 | *P* = 0.207 |
| Perioperative mortality | 7 | OR = 2.29 | 0.94-5.57 | I^2^ =14.5 %, *P* = 0.319 | *P* = 0.067 |
| Intraoperative blood loss | 5 | SMD = -0.50 | -0.82 to -0.18 | I^2^ =68.0 %, *P* = 0.014 | *P* = 0.002 |
| Total hospital stay | 7 | WMD = -4.87 | -7.34 to -2.41 | I^2^ = 81.7%, *P* < 0.001 | *P* < 0.001 |
| 5-year DFS | 2 | HR = 1.56 | 0.75-3.26 | I^2^ =67.7 %, *P* = 0.078 | *P* = 0.234 |
| 5-year OS | 5 | HR = 1.11 | 0.88-1.40 | I^2^ = 0.0%, *P* = 0.601 | *P* = 0.366 |
| **More than 50 patients in the simultaneous group** |  |  |  |  |  |
| Total complications | 7 | OR = 1.05 | 0.76-1.46 | I^2^ =59.7 %, *P* =0.021 | *P* = 0.769 |
| Gastrointestinal complications | 7 | OR = 1.09 | 0.74-1.59 | I^2^ =47.6%, *P* = 0.075 | *P* = 0.673 |
| Hepatic complications | 7 | OR = 1.14 | 0.88-1.48 | I^2^ =0.0 %, *P* = 0.834 | *P* = 0.315 |
| Perioperative mortality | 6 | OR = 1.00 | 0.44-2.24 | I^2^ = 0.0%, *P* = 0.760 | *P* = 0.990 |
| Intraoperative blood loss | 6 | WMD = -239.90 | -358.12 to -121.68 | I^2^ = 88.0%, *P* < 0.001 | *P* < 0.001 |
| Total hospital stay | 5 | WMD = -1.17 | -1.67 to -0.68 | I^2^ = 89.5%, *P* < 0.001 | *P* < 0.001 |
| 5-year DFS | 2 | HR = 1.14 | 0.83-1.56 | I^2^ =0.0 %, *P* = 0.956 | *P* = 0.412 |
| 5-year OS | 5 | HR = 1.16 | 0.88-1.52 | I^2^ = 63.3%, *P* = 0.028 | *P* = 0.302 |

**Table S1** The results of all subgroup analyses
